# Upregulation of alveolar fluid clearance is not sufficient for Na^+^,K^+^-ATPase β subunit-mediated gene therapy of LPS-induced acute lung injury in mice

**DOI:** 10.1038/s41598-023-33985-4

**Published:** 2023-04-26

**Authors:** Jing Liu, Gillian M. Schiralli-Lester, Rosemary Norman, David A. Dean

**Affiliations:** 1 Department of Pediatrics, University of Rochester, 601 Elmwood Avenue, Box 850, Rochester, NY 14642 USA; 2 Department of Pharmacology and Physiology, University of Rochester, 601 Elmwood Avenue, Rochester, NY 14642 USA

**Keywords:** Respiration, Gene therapy

## Abstract

Acute Lung Injury/Acute Respiratory Distress Syndrome (ALI/ARDS) is characterized by diffuse alveolar damage and significant edema accumulation, which is associated with impaired alveolar fluid clearance (AFC) and alveolar‐capillary barrier disruption, leading to acute respiratory failure. Our previous data showed that electroporation‐mediated gene delivery of the Na^+^, K^+^-ATPase β1 subunit not only increased AFC, but also restored alveolar barrier function through upregulation of tight junction proteins, leading to treatment of LPS‐induced ALI in mice. More importantly, our recent publication showed that gene delivery of MRCKα, the downstream effector of β1 subunit-mediated signaling towards upregulation of adhesive junctions and epithelial and endothelial barrier integrity, also provided therapeutic potential for ARDS treatment in vivo but without necessarily accelerating AFC, indicating that for ARDS treatment, improving alveolar capillary barrier function may be of more benefit than improving fluid clearance. In the present study, we investigated the therapeutical potential of β2 and β3 subunits, the other two β isoforms of Na^+^, K^+^-ATPase, for LPS‐induced ALI. We found that gene transfer of either the β1, β2, or β3 subunits significantly increased AFC compared to the basal level in naïve animals and each gave similar increased AFC to each other. However, unlike that of the β1 subunit, gene transfer of the β2 or β3 subunit into pre-injured animal lungs failed to show the beneficial effects of attenuated histological damage, neutrophil infiltration, overall lung edema, or increased lung permeability, indicating that β2 or β3 gene delivery could not treat LPS induced lung injury. Further, while β1 gene transfer increased levels of key tight junction proteins in the lungs of injured mice, that of either the β2 or β3 subunit had no effect on levels of tight junction proteins. Taken together, this strongly suggests that restoration of alveolar-capillary barrier function alone may be of equal or even more benefit than improving AFC for ALI/ARDS treatment.

## Introduction

Acute Respiratory Distress Syndrome (ARDS) is a devastating clinical condition of acute respiratory failure^1^. It is characterized by the accumulation of pulmonary edema of noncardiogenic origin and diffuse alveolar damage (DAD) that is primarily caused by alveolar capillary barrier dysfunction and the resulting alveolar and interstitial flooding of protein rich fluid^1,2^. It is estimated that there are 190,000 cases of ARDS annually in the United States with a hospital mortality of up to 40%^3^. During the evolving COVID-19 pandemic, approximately 33% of hospitalized COVID-19 patients develop ARDS, and the incidence of ARDS among non-survivors of COVID-19 reaches up to 90%, indicating that ARDS accounts for the majority of COVID-19 deaths^4^. Even so, the development of pharmacological treatments for ARDS has lagged since there is no single gene mutation or mechanism responsible for ARDS. Clinical strategies so mainly rely on supportive care and ventilatory management^5^.

Increasing alveolar fluid clearance (AFC) has been one primary therapeutic targets to treat ARDS based on the finding that there is impaired fluid clearance capacity and significant edema accumulation in the lung during early ARDS^6,7^. AFC is driven by the osmotic gradient which is created by active ion transport, primarily Na^+^, across the alveolar epithelium^8^. Consequently, fluid is moved out from the airspace into lung interstitium along with the Na^+^ flux. Many researchers have focused on overexpressing or upregulating ion channels or transporters in the lung to increase AFC, such as the Na^+^,K^+^-ATPase, ENaC, and CFTR^7,9–11^. Pharmacologically, β_2_ adrenergic agonists have been administered to enhance AFC in various animal models by increasing the activity or membrane abundance of ENaC or the Na^+^,K^+^-ATPase^12^. However, this strategy has failed in clinical trials and shown no significant improvement of mortality rate^13^. Gene delivery mediated direct overexpression of ENaC or CFTR into living lungs also induces the increased transepithelial movement of Na^+^ towards the interstitium and therefore, enhances AFC. When tested in animal models of acute lung injury, the benefit of increasing AFC could easily be seen when the target genes were delivered prior to induction of lung injury, indicating a protective effect, but had little if any effect when delivered after injury had been established, suggesting that these may not be viable treatment approaches^14,15^.

In studying the therapeutical potential of gene delivery of Na^+^,K^+^-ATPase subunits for treating ARDS, our lab and others found that overexpression of Na^+^,K^+^-ATPase β1 subunit into lungs not only augmented AFC, protecting animals against the subsequent lung injury, but also rescued mice from pre-existing established injury induced by endotoxin^7,16^. Functionally, the Na^+^,K^+^-ATPase is critical to AFC, and more importantly, it is closely involved in the regulation of the epithelial barrier integrity^17^. Underlying ARDS, the net fluid clearance responsible for edema clearance from the airspace is undermined not by the impaired AFC, but also by alveolar capillary barrier dysfunction^6^. Our previous data showed that electroporation mediated gene transfer of the Na^+^,K^+^-ATPase β1 subunit could treat previously injured lungs, showing significantly upregulated tight junction protein expression, decreased lung permeability, edema accumulation, total protein and cellularity in bronchoalveolar lavage (BAL) fluid, and improved overall outcome of lung injury^14,18^. While these experiments showed that gene transfer of ENaC actively gave higher rates of AFC than did that of the Na^+^,K^+^-ATPase β1 subunit, only the β1 subunit had any treatment effect in previously injured lungs, indicating the significance of repairing alveolar capillary barrier integrity for ARDS resolution. Moreover, we recently demonstrated that CDC42 binding protein kinase alpha (MRCKα), a downstream mediator of β1 subunit-mediated signaling to upregulate tight junction and epithelial barrier integrity in vitro, also provided therapeutic potential for ARDS treatment in vivo^18,19^. Overexpression of MRCKα attenuated LPS increased pulmonary edema (Wet/Dry ratio), lung leakage, inflammation, and improved overall outcome of lung injury all to the same degree as seen with β1 gene transfer, but without improving AFC. These results further suggest that restoring lung barrier function alone may be of equal or even more benefit than improving AFC for treating ARDS.

To date, three β isoforms, β1, β2, and β3, have been identified as being expressed in mammalian cells^20–22^. Although there is isoform selectivity, all of them are able to bind to the α subunit forming a functional pump for Na^+^ transport^20,23^. The α1β1 isozyme functions as a housekeeping Na^+^,K^+^-ATPase in all cells, vital for maintaining the intracellular ion homeostasis, and is highly conserved across species^23^. The regulatory role of the β1 subunit for epithelial barrier integrity is also conserved from insects to vertebrates^24^. For example, Nrv2, the vertebrate β1 homologue, is the only isoform involved in the epithelial septate junction barrier function in *Drosophila*^25^. Other β subunit isoforms are tissue-specific: the β2 isoform is expressed predominantly in brain and muscle, and the β3 isoform is found in lung, testis, skeletal muscle, and liver^23^. Sequence alignment shows there is only 25% homology among the three transport functional β isoforms with high diversity in their extracellular domains, including their degree of glycosylation. Our previous in vitro data showed that contrary to the gene transfer of the β1 subunit, gene delivery of the β2 or β3 subunit to cultured alveolar epithelial cells failed to induce tight junction expression in a monolayer^19^. Thus, in the present study, we tested whether overexpression of the β2 or β3 subunit of the Na^+^, K^+^-ATPase, like the β1 subunit, could upregulate pulmonary barrier function in vivo and be used to treat mouse lungs previously injured with LPS. Understanding this will help further evaluate the relative importance and contribution of lung barrier integrity and alveolar fluid clearance in the development of treatments for ARDS.

## Materials and methods

### Plasmids

Plasmid pcDNA3 was obtained from Promega (Madison, WI, USA). Plasmids pCMV6-Na^+^, K^+^-ATPase β1, β2, and β3 expressing Myc-DDK-tagged human Na^+^, K^+^-ATPase β1, β2, β3 subunits respectively, were purchased from Origene (Rockville, MD, USA). Plasmids were purified using Qiagen Giga-prep kits (Qiagen, Chatsworth, CA, USA) and suspended in 10 mM Tris–HCl (pH 8.0), 1 mM ethylenediaminetetraacetic acid (EDTA) and 140 mM NaCl.

### In vivo gene transfer and induction of acute lung injury (ALI)

ALI was induced in male C57BL/6 mice (8–10 weeks) by oropharyngeal aspiration of 50 ul of Escherichia coli 055:B5 LPS (5 mg/kg; Sigma-Aldrich, St. Louis, MO, USA) as described previously^14,18^. Twenty-four hours later, mice were anesthetized with isoflurane (2–4%) and 100 μg of plasmid in 50 μl of 10 mM Tris–HCl (pH 8.0), 1 mM EDTA and 140 mM NaCl was delivered to mouse lungs by oropharyngeal aspiration and electroporation with eight 10-ms square wave pulses at field strength of 200 V cm^−1^ using externally placed pediatric pacemaker electrodes (Medtronic, Redmond, WA, USA) on both sides of the chest with an ECM830 electroporator (BTX, Harvard Apparatus, Holliston, MA, USA) as described^14,18^. Each animal received one plasmid only (e.g., pCMV-β1, pCMV-β2, or pCMV-β3) as opposed to combinations of the plasmids. Measures of lung injury were assessed 2 days after gene delivery (n = 6–8 mice/group). All experiments were repeated three independent times. To ensure that oropharyngeal aspiration resulted in plasmid delivery primarily to the lung, we delivered 50 µl of Evans Blue dye-labeled albumin to the lungs of mice and then isolated the lungs and the esophagus/stomach within 5 min of aspiration prior to drying and spectrophotometrically quantifying Evans Blue dye. As can be seen in the Fig. S1, greater than 92% of the Evans Blue was localized in the lungs and less than 8% was detected in the GI tract.

### Western blot analysis

Western blot was performed using half of the left lung lobe that was homogenized in 300 μl of 1× Reporter lysis buffer (Promega, Madison, WI, USA), containing protease inhibitor (Roche, Basel, Switzerland) using a TissueLyser II (Qiagen, Germantown, MD, USA) as previously described^14^. Following SDS-PAGE and transfer to PVDF membrane, blots were probed with primary antibodies against DDK tag (Origene, Rockville, MD, USA), ZO-1 (Invitrogen, Rockford, IL, USA), occludin (Invitrogen), VE-cadherin (Invitrogen, Rockford, IL, USA), and glyceraldehyde-3-phosphate dehydrogenase GAPDH (EMD Millipore, Burlington, MA, USA). Data were analyzed using the NIH Image J software.

### Measurement of wet to dry ratios

Wet to dry (W/D) ratios, as a measure of pulmonary edema, were measured 72 h after LPS instillation as previously described^14,18^. Briefly, lungs were removed from the mice and weighed immediately (wet weight) prior to drying them for 72 h at 70 °C and weighing again (dry weight).

### BAL analysis

Protein concentration and cellularity of BAL fluid was measured as described previously^18,26^. Briefly, 0.7 ml of sterile PBS was instilled into mouse lungs, lavaged twice, collected and centrifuged. Total protein concentration of the supernatant was measured using a Bradford assay (Bio-Rad), and the total number of cells was determined using a hemocytometer and then cells were stained with Diff-Quick (Siemens, Newark, DE, USA) after cytospin. Albumin levels in BAL fluid of each group was assessed using a mouse albumin ELISA quantitation kit (Bethyl Laboratories, Montgomery, TX) as described^18^.

### Histological analysis

Lungs were inflated to a pressure of 20 cm H_2_O with buffered formalin, removed from the chest, fixed overnight at 4 °C and paraffin-embedded. Sections (5 µm) were stained with hematoxylin and eosin (H & E).

### Lung permeability assay

Pulmonary permeability was performed as described to assess alveolar capillary barrier integrity by the leakage of Evans Blue Dye (EBD) labeled albumin from blood into airways^14,18^. Briefly, EBD (30 mg kg^−1^, Sigma) was injected into the tail vein 47 h after gene transfer and the lungs were perfused 1 h later with sterile PBS, excised, and dried at 60 °C for 24 h. EBD was quantified spectrophotometrically at 620 and 740 nm, and corrected with the formula E_620_ (EBD) = E_620_ − (1.426 × E_740_ + 0.030).

### Measurement of AFC in live mice

AFC was evaluated by quantifying the rate of concentration of an isosmolar (324 mOsm) solution of EBD labeled albumin in the alveolar airspace as previously described^18^. Procaterol (a specific β2AR agonist, 10^−8^ mol/L) was administered in the instillate as a positive control.

### Statistical analysis

Each experiment was repeated at least three times with the number of animals per group indicated for each figure (n = 6–14). The data of each series is displayed as mean values ± standard error of the mean (SEM). Graphing and statistical comparison of the data were performed using Prism 8 (GraphPad Software, San Diego, CA, USA). Measurements for more than two groups were analyzed by one-way ANOVA followed by multiple comparisons. P values less than 0.05 were considered to be statistically significant.

### Study approval

All animal studies were approved by the University of Rochester Committee on Animal Resources and experimental procedures were carried out under the institutional guidelines for the care and use of laboratory animals in an American Association for the Accreditation of Laboratory Animal Care-approved facility. All animal studies complied with the ARRIVE guidelines.

## Results

### Electroporation mediated gene transfer of β1, β2, or β3 subunits of the Na^+^,K^+^-ATPase upregulates alveolar fluid clearance in mice

**Figure 1:**
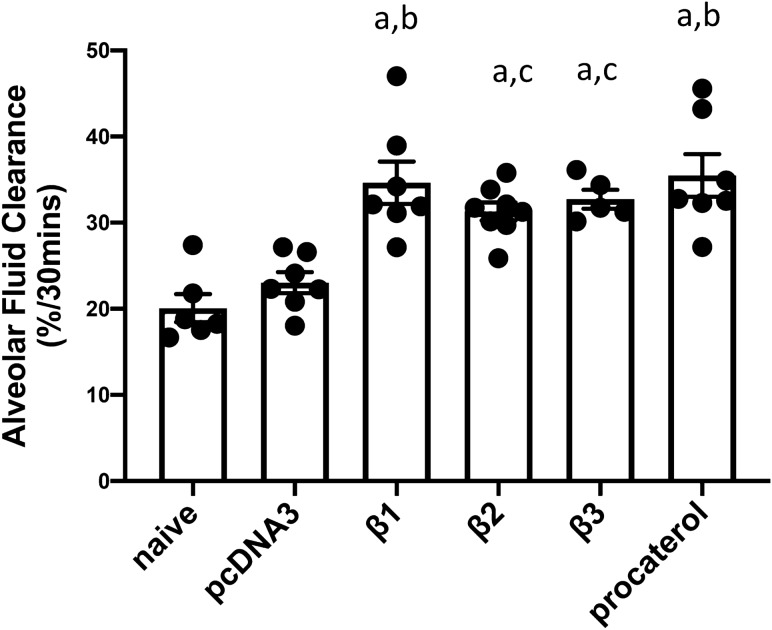
Electroporation mediated gene transfer of β1, β2, and β3 subunits of the Na^+^,K^+^-ATPase upregulate alveolar fluid clearance in mice. Plasmids (100 μg each) expressing either no insert (pcDNA3), or the β1, β2, or β3 subunits of the Na^+^,K^+^-ATPase (β1, β2, or β3) were delivered in 50 μl to the lungs of C75B6 mice (n = 6–7) by aspiration followed immediately by electroporation (8 pulses of 10 ms duration each and 200 V/cm). Naïve mice received no DNA. Two days later, alveolar fluid clearance was measured in living mice and calculated based on the change in concentration of Evans Blue Dye-labeled albumin in an isosmolar (324 mOsm) instillate placed into the alveolar space and mechanically ventilated over a 30 min period. Procaterol (10^–8^ mol/L) was administered in the instillate and used as the positive control in a set of naïve mice. Rates of alveolar fluid clearance are shown as mean ± SEM. All experiments were carried out three times and a representative experiment is shown. One-way ANOVA with post-hoc Tukey’s multiple comparisons was used for statistical analysis; a, p < 0.001 compared to naïve; b, p < 0.01 compared to pcDNA3; c, p < 0.05 compared to pcDNA3.

Fluid reabsorption from the alveolar space has been well documented based on the osmotic pressure created by transepithelial active ion transport, primarily Na^+^ transport^6,17^. Extensive research in animal models and patients has shown that upregulating Na^+^,K^+^-ATPase activity or its abundance through β adrenergic stimulation or gene delivery of the Na^+^,K^+^-ATPase α1 or β1 subunits significantly enhances alveolar fluid clearance (AFC) from its basal level, indicating there is increased active Na^+^ transport activity. β2 and β3, two additional β isoforms, are expressed in a tissue- and cell-specific manner to form active Na^+^ pumps for ion extrusion^20^. To further validate that gene transfer of the β2 and β3 subunits could also accelerate alveolar fluid clearance by overexpressing Na^+^,K^+^-ATPase and thereby increasing Na^+^ absorption from alveoli, genes encoding the β2 and β3 subunits were delivered to mouse lungs by electroporation and 2 days later, AFC was measured in living lungs (Fig. 1). Electroporation-mediated gene transfer of all three DDK-tagged beta subunits resulted in significant overexpression in the mice (Figs. S2 and S3). Gene transfer of β2- or β3-Na^+^, K^+^-ATPase significantly enhanced AFC (31.32 ± 1.042 for β2, 32.73 ± 1.097 for β3) by 55% and 63% compared with naïve animals (20.08 ± 1.64, p < 0.01) and mice that received empty plasmid pcDNA3 (20.04 ± 1.21, p < 0.001) by 54% and 62%, respectively. There was no significant difference in AFC rates between naïve and pcDNA3 mice. Animals receiving either β1 gene transfer or procaterol (10^−8^ mol/L), a specific β2-Adrenergic receptor agonist, were used as positive controls, leading to increases of AFC by 73% and 78%, respectively, compared with naïve mice (p < 0.01). There was no significant difference in AFC between animals receiving the β1, β2, or β3 subunits, indicating that all 3 subunits can increase AFC to similar degrees. Further, the significantly increased AFC from the naive basal level caused by β2 or β3 overexpression suggests that these two β isoforms can heterodimerize with the endogenous α subunit to form functional Na^+^,K^+^-ATPases for ion transport in the lung.

### Gene delivery of β2 or β3 subunits of the Na^+^,K^+^-ATPase fails to attenuate inflammation in LPS injured lungs

**Figure 2:**
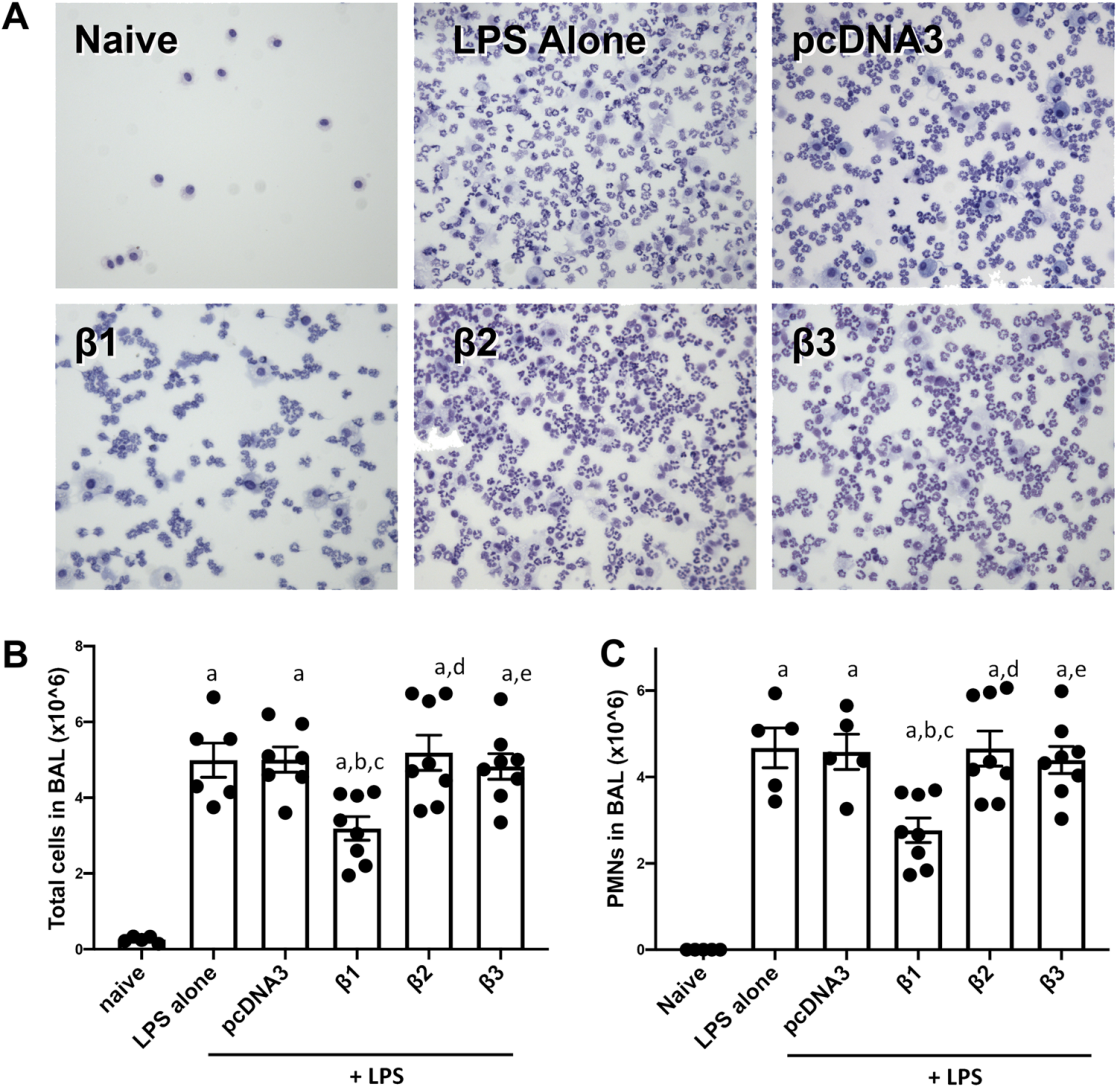
Electroporation mediated gene delivery of the β1, but not the β2 or β3 subunits, of the Na^+^,K^+^-ATPase attenuates inflammation in LPS injured lungs. Lung injury was established in C57B6 mice (n = 6–8) by aspiration of LPS (5 mg/kg) and 1 day later plasmids (100 μg each) expressing either no insert (pcDNA3), or the β1, β2, or β3 subunits of the Na^+^,K^+^-ATPase (β1, β2, or β3) were delivered in 50 μl to the lungs by aspiration followed immediately by electroporation (8 pulses of 10 ms duration each and 200 V/cm). Naïve mice (n = 5) received no LPS or DNA. Two days after gene transfer (3 days after LPS administration), lungs were lavaged with PBS and BAL fluid was collected and analyzed for cellularity by cytospin followed by Diff-quik staining (**A**). All experiments were carried out three times and a representative experiment is shown. Total cells were quantified in the BAL fluid and shown as mean ± SEM (**B**). One-way ANOVA with post-hoc Tukey’s multiple comparisons was used for statistical analysis; a, p < 0.0001 compared to naïve; b, p < 0.05 compared to LPS alone; c, p < 0.05 compared to LPS + pcDNA3; d, p < 0.01 compared to LPS + β1; e, p < 0.05 compared to LPS + β1. The number of PMNs in the BAL fluid were also quantified and shown as mean ± SEM (**C**). One-way ANOVA with post-hoc Tukey’s multiple comparisons was used for statistical analysis; a, p < 0.0001 compared to naïve; b, p < 0.01 compared to LPS alone; c, p < 0.05 compared to LPS + pcDNA3; d, p < 0.01 compared to LPS + β1; e, p < 0.05 compared to LPS + β1.

We previously have reported that electroporation mediated gene transfer of β1-Na^+^,K^+^-ATPase can effectively attenuate LPS-induced acute lung injury in vivo with decreased total cellularity and PMNs in BAL fluid, compared with LPS injury alone^14^. This has been seen in both protection experiments where β1 is transferred prior to lung injury and in the more stringent and clinically relevant treatment model where DNA is delivered to lungs with established lung injury. Since gene delivery of the β2 or β3 subunit of the Na^+^,K^+^-ATPase could also enhance AFC to the same degree as that by β1 overexpression, we next determined whether gene delivery of β2 or β3 subunits could also attenuate inflammation in LPS pre-injured animal lungs. BAL fluid was harvested from mouse lungs and prepared for total cell counting and differential cell staining. Since PMNs are the predominant immune cell type in BAL fluid, PMNs were also counted for statistical analysis (Fig. 2). Consistent with our previous results, instillation of LPS significantly increased immune cell infiltration (4.992 ± 0.451, p < 0.0001) and predominantly PMNs (4.673 ± 0.461, p < 0.0001), compared with healthy mice (0.255 ± 0.036 for total cells; 0.001 for PMNs). There was no significant difference in cellularity (total cells or PMNs) between LPS alone and LPS followed by the empty vector pcDNA3 group. Mice electroporated with plasmid encoding β1-Na^+^,K^+^-ATPase showed the significant reduction in the number of total cells (3.188 ± 0.311, p < 0.05) and PMNs (2.767 ± 0.284, p < 0.01) in BAL fluid, compared with LPS injury alone. Surprisingly, gene transfer of the β2 or β3 subunit of the Na^+^,K^+^-ATPase into LPS pre-injured animal lungs did not decrease the total number of cells (5.188 ± 0.464 for β2 plasmid; 4.825 ± 0.339 for β3 plasmid) or PMNs (4.659 ± 0.405 for β2 plasmid; 4.394 ± 0.314 for β3 plasmid)) in BAL fluid, and there was no statistical difference between β2 or β3 overexpression mice and LPS injury group (not shown). Further, there was no difference in cell numbers between mice receiving β2 or β3 plasmid and animals receiving empty vector. Collectively, these results indicate that unlike the β1 subunit, electroporation mediated gene transfer of β2 or β3 Na^+^,K^+^-ATPase failed to attenuate LPS induced lung inflammation.

### Overexpression of β1-Na^+^,K^+^-ATPase, but not β2 or β3, attenuates the overall lung injury in LPS injured living lungs

**Figure 3:**
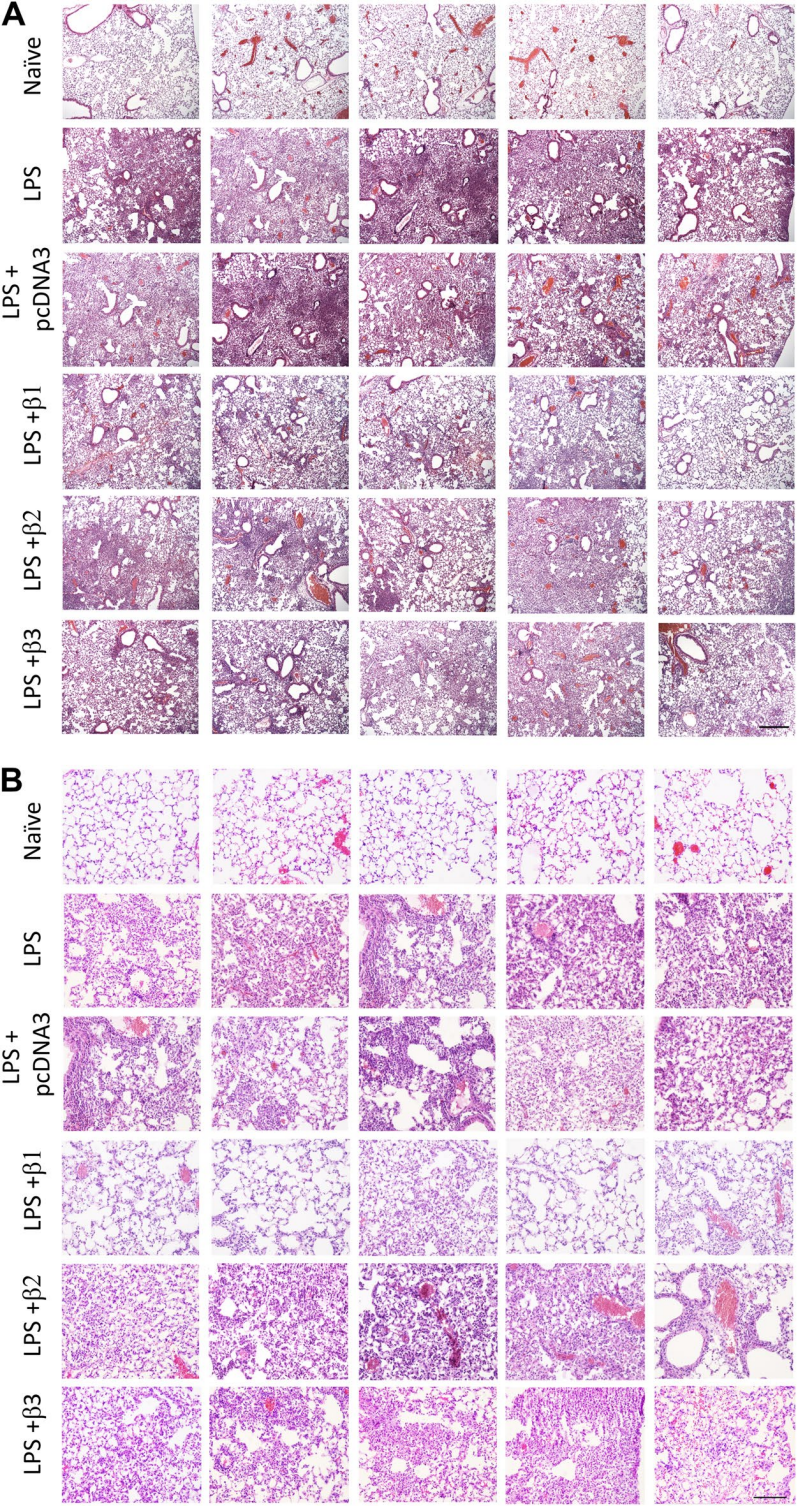
Overexpression of the β2 or β3 subunits of the Na^+^,K^+^-ATPase fails to attenuate LPS-induced lung injury. Lung injury was established in mice (n = 6–8) by aspiration of LPS (5 mg/kg) and 1 day later plasmids (100 μg each) expressing either no insert (pcDNA3), or the β1, β2, or β3 subunits of the Na^+^,K^+^-ATPase (β1, β2, or β3) were electroporated into lungs as in Fig. 2. Naïve mice (n = 5) received no LPS or DNA. Two days after electroporation (3 days after LPS administration), lungs were inflated to 20 cm H_2_O with 10% buffered formalin and processed for paraffin-embedding, sectioning, and hematoxylin and eosin staining. Sections from 5 representative animals are shown at ×50 (**A**) and ×400 (**B**) magnification. All experiments were carried out three times and a representative experiment is shown. Scale bar is 300 μm (**A**) and 100 μm (**B**).

We next looked at overall lung injury by histological analysis to determine whether gene delivery of β2 or β3 subunits could attenuate LPS induced injury. Figure 3 shows random images taken of 5 different mice from each treatment cohort at low and high magnification. As expected, compared with healthy lungs which show intact alveolar architecture, clear airspaces, thin alveolar walls with occasional infiltrating cells, and no evidence of pulmonary edema or fibrin deposition, lungs injured with LPS alone or LPS followed 1 day later with the empty plasmid pcDNA3 showed significant atelectasis, infiltrating cells (primarily PMNs), as well as evident proteinaceous edema and thickened alveolar walls. Gene transfer of the Na^+^,K^+^-ATPase β1 subunit showed a therapeutic effect on the injured lungs, notably reducing patchy areas of neutrophil infiltration, alveolar edema and deposited fibrin strands, and showing increased numbers of areas of open and clear alveoli. However, gene delivery of the β2 or β3 subunits failed to show noticeable attenuation of any of these hallmarks of diffuse alveolar damage, compared with the LPS injury group or pcDNA3. Taken together, all the data here indicate that gene transfer of β1-Na^+^,K^+^-ATPase provides a therapeutic benefit attenuating lung inflammation and overall damage, whereas gene delivery of β2 or β3 isoforms of the Na^+^, K^+^-ATPase has no treatment effect on lung injury.

### Gene transfer of β2 or β3 subunits fails to reduce lung edema.

**Figure 4:**
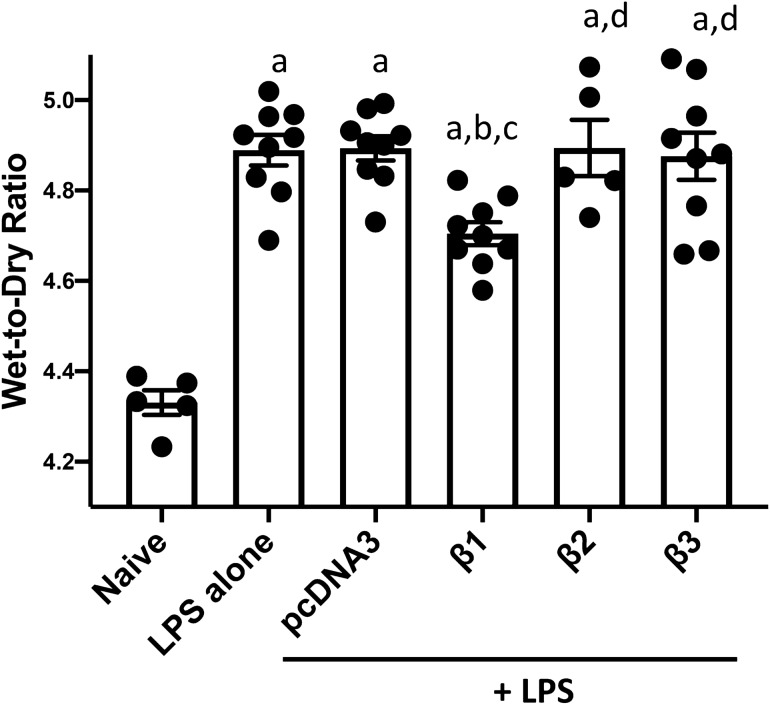
Gene transfer of β2 or β3 subunits of the Na^+^,K^+^-ATPase into mouse lungs with existing LPS-induced injury failed to reduce lung edema. Lung injury was established in C57B6 mice (n = 9) by aspiration of LPS (5 mg/kg) and 1 day later plasmids (100 μg each) expressing either no insert (pcDNA3), the β1, β2, or β3 subunits of the Na^+^,K^+^-ATPase (β1, β2, or β3) were delivered in 50 μl to the lungs by aspiration followed immediately by electroporation. Two days later, wet to dry ratios were determined as a measure of pulmonary edema fluid and shown as mean ± SEM. All experiments were carried out three times and a representative experiment is shown. One-way ANOVA with post-hoc Tukey’s multiple comparisons was used for statistical analysis; a, p < 0.0001 compared to naïve; b, p < 0.05 compared to LPS alone; c, p < 0.01 compared to LPS + pcDNA3; d, p < 0.05 compared to LPS + β1.

One feature characterizing diffused alveolar damage is the alveolar flooding to form edema at the early stage of ARDS. We investigated whether gene transfer of the β2 or β3 subunit could reduce the general edema fluid retention following lung injury, since they markedly increase AFC following gene transfer in mice (Fig. 1). As above, 1 day after LPS administration, plasmids encoding β2-, or β3-Na^+^,K^+^-ATPase were delivered to mouse lungs and 2 days later, lungs were harvested for gravimetric analysis (Fig. 4). Compared with the naïve group ((4.331 ± 0.027), mice injured with LPS alone or receiving pcDNA3 following injury showed increased wet to dry ratios of4.889 ± 0.034 (p < 0.001) for LPS alone and 4.893 ± 0.027 (p < 0.001) for LPS with pcDNA3, respectively, indicating significant edema accumulation in the lung. Consistent with previously published studies, gene transfer of β1-Na^+^, K^+^-ATPase, which was used as positive control, significantly reduced LPS induced lung edema by ~ 27% with wet to dry ratio of 4.705 ± 0.025, compared with LPS alone (p < 0.05), or LPS plus empty vector(p < 0.01)^14,18,26,27^. However, the injured lungs receiving plasmids to overexpress the β2 or β3 subunit of Na^+^, K^+^-ATPase failed to show any changes in their wet to dry ratios (4.894 ± 0.062 for LPS with β2, 4.876 ± 0.052 for LPS with β3), compared with injury groups (LPS alone or LPS plus pcDNA3), and there was no statistical significance between treatment groups of LPS with β2 Na^+^,K^+^-ATPase, or β3Na^+^, K^+^-ATPase and mice with LPS alone or LPS plus pcDNA3.

### Electroporation mediated gene transfer of the β1, but not β2 or β3 subunit, of the Na^+^,K^+^-ATPase attenuates LPS increased lung permeability in mice

**Figure 5:**
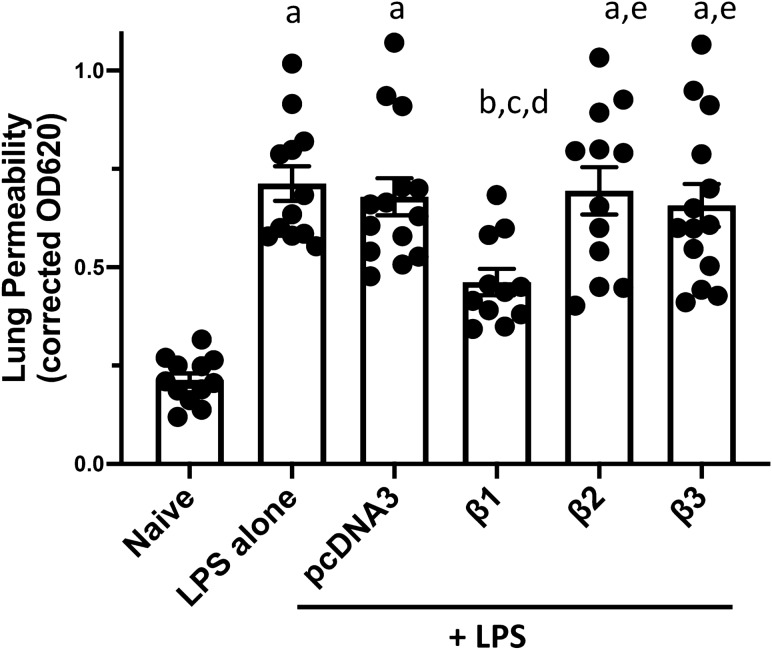
Electroporation mediated gene transfer of the β1, but not the β2 or β3, subunit of the Na^+^,K^+^-ATPase attenuates LPS increased lung permeability in mice. Lung injury was established in C57B6 mice (n = 11–14) followed 1 day later by gene transfer as described in Fig. 2. Forty-seven hours after gene transfer, Evans Blue Dye (30 mg/kg) was administered by tail vein injection and one hour later, lungs were perfused with PBS and harvested for dye extraction. Lung permeability was evaluated by quantifying the absorbance of extracted Evans Blue Dye and shown as mean ± SEM. All experiments were carried out three times and a representative experiment is shown. One-way ANOVA with post-hoc Tukey’s multiple comparisons was used for statistical analysis; a, p < 0.0001 compared to naïve; b, p < 0.01 compared to naïve; c, p < 0.01 compared to LPS alone; d, p < 0.05 compared to LPS + pcDNA3; e, p < 0.05 compared to LPS + β1.

The gravimetric analysis of wet to dry weight ratio is a gross indicator of lung edema accumulation, reflecting the result of net fluid clearance which is composed of both the osmotic driving force for alveolar fluid absorption and an intact alveolar capillary barrier which holds out against alveolar flooding^6^. Since gene delivery of the β2 or β3 subunit of the Na^+^,K^+^-ATPase could accelerate AFC but failed to decrease or partially reverse the LPS increased edema accumulation, we further determined the injured animals’ lung barrier function after treatment intervention with the various β subunits. Thereby, in vivo lung permeability was determined by the leakage of EBD labeled albumin from blood into airways (Fig. 5)^14^. Forty-seven hours after delivering plasmids encoding pcDNA3, β1-, β2- and β3-Na^+^,K^+^-ATPase, respectively, to LPS pre-injured mouse lungs, the EBD labeled albumin was injected into circulation via the tail vein and 1 h later, lungs were harvested for EBD extraction. The more EBD extracted, the more extravasation of EBD leaking into lung tissue, indicating severe alveolar capillary barrier disruption and higher alveolar-capillary barrier permeability. As described above, compared with the naïve group (0.213 ± 0.017), mice injured with LPS alone showed an up to threefold increase of spectrophotometric absorbance of EBD (0.713 ± 0.044), indicating severe lung barrier leakage (p < 0.0001). Gene delivery of empty vector pcDNA3 (0.679 ± 0.047) showed no significant difference compared to LPS alone. As expected, gene transfer of β1-Na^+^, K^+^-ATPase, the positive control, significantly reversed the LPS increased lung permeability by more than 30% (0.462 ± 0.033), compared to LPS alone or LPS plus pcDNA3, indicating the significant rescue of lung barrier function (p < 0.01 and p < 0.05, respectively). However, gene delivery of β2 or β3 subunits failed to attenuate LPS increased lung permeability with EBD spectrophotometric absorbance of 0.694 ± 0.060 for the LPS with β2 group and 0.657 ± 0.055 for the LPS with β3 group, indicating there was still severe lung leakage in β2 or β3 treated mice.

### Gene transfer of the β2 or β3 subunit fails to reduce BAL protein levels in previously injured mice

**Figure 6:**
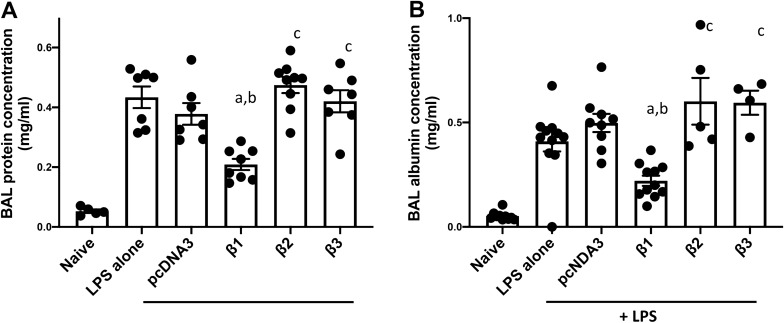
Gene transfer of the β2 and β3 subunits of the Na,K-ATPase fail to reduce protein levels in the BAL in LPS-injured lungs. Lung injury was established in C57B6 mice (n = 6–8) by aspiration of LPS (5 mg/kg) and 1 day later plasmids (100 μg each) expressing either no insert (pcDNA3), the β1, β2, or β3 subunits of the Na^+^,K^+^-ATPase (β1, β2, or β3) were delivered in 50 μl to the lungs by aspiration followed immediately by electroporation. Two days after gene delivery, lungs were lavaged with PBS and BAL fluid was collected and analyzed for total protein (**A**) or serum albumin (**B**). All experiments were carried out three times and a representative experiment is shown. One-way ANOVA with post-hoc Tukey’s multiple comparisons was used for statistical analysis; For total protein (**A**) a, p < 0.001 compared to LPS alone; b, p < 0.01 compared to LPS + pcDNA3; c, p < 0.001 compared to LPS + β1. For Albumin (**B**) a, p < 0.001 compared to LPS alone; b, p < 0.001 compared to LPS + pcDNA3; c, p < 0.001 compared to LPS + β1.

Since gene delivery of β2- or β3-Na^+^, K^+^-ATPase failed to attenuate LPS induced lung hyperpermeability, we further examined the total protein and high molecular weight protein albumin level in BAL fluid of injured mice, which are other parameters reflecting the alteration of alveolar capillary barrier. Mice injured with LPS significantly increased the total protein (0.434 ± 0.036, p < 0.0001, Fig. 6A) and specific albumin level (0.410 ± 0.049, p < 0.0001, Fig. 6B) in BAL, compared with the naïve group, indicating severe LPS induced microvascular and alveolar epithelial leakage. The concentration of total protein (0.378 ± 0.036, Fig. 6A) or albumin (0.498 ± 0.043, Fig. 6B) in BAL fluid of mice with LPS and pcDNA3 is not significantly different from that of LPS mice. Compared with mice with LPS injury alone or with empty vector, transfer of the β1 plasmid to injured mouse lungs significantly reduced the concentration of total protein (0.209 ± 0.019, p < 0.001 for LPS injury alone, p < 0.01 for LPS with pcDNA3), as well as serum albumin (0.221 ± 0.024, p < 0.001) in the BAL, indicate the pulmonary barrier restoration by β1 gene therapy. In contrast, gene delivery of β2-, or β3-Na^+^, K^+^-ATPase failed to decrease the total protein (0.475 ± 0.027 for LPS with β2, 0.421 ± 0.037 for LPS with β3) and albumin (0.602 ± 0.112 for LPS with β2, 0.595 ± 0.058 for LPS with β3) level observed in mice with LPS injury alone or with following pcDNA3, further indicating there is no restoration or rescue of the lung barrier hyperpermeability and leakage by transferring genes of β2 or β3.

### Restoration of ZO-1, occludin, and VE-cadherin expression in previously LPS injured mouse lungs by gene transfer of the β1 subunit

**Figure 7:**
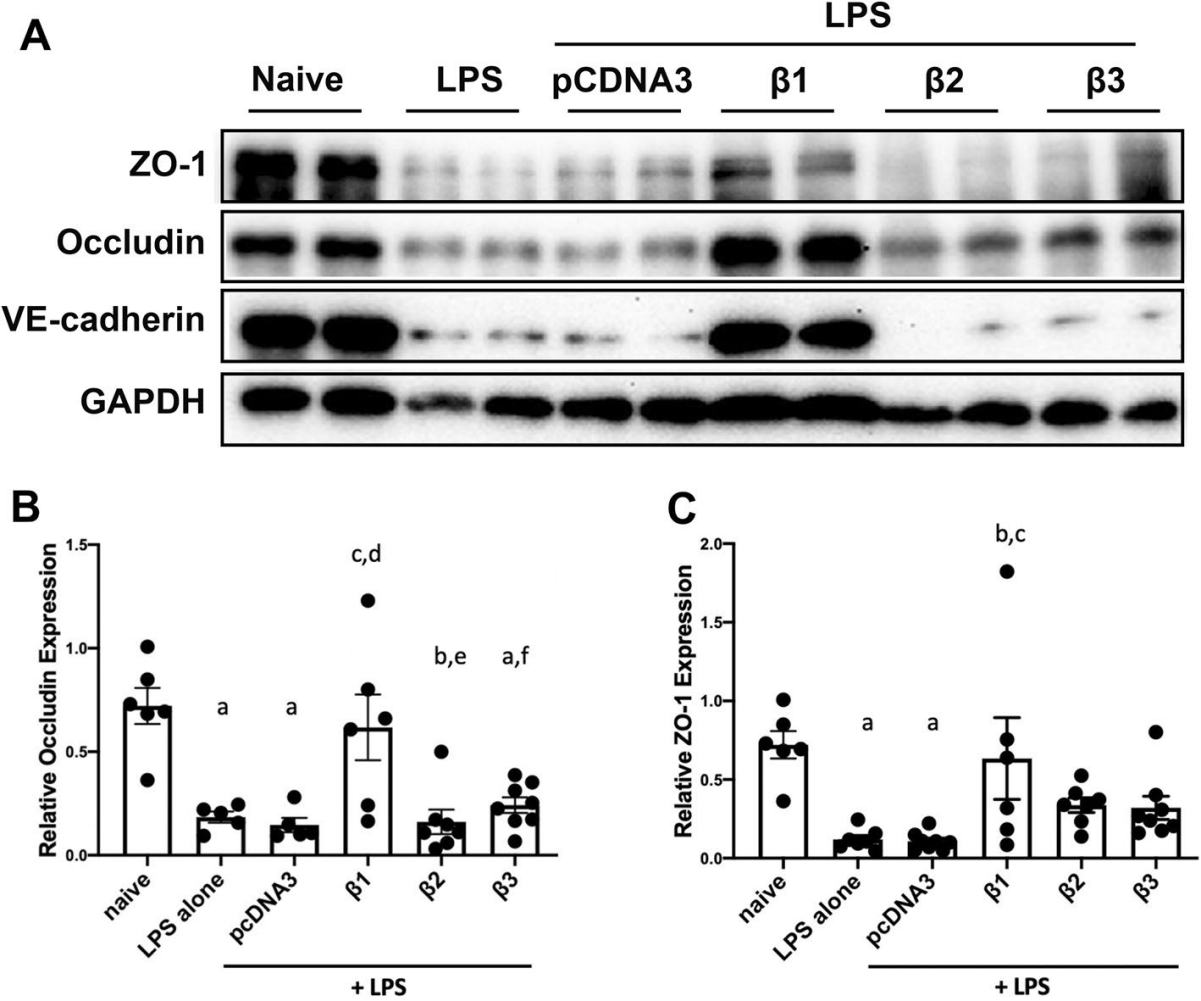
Gene transfer of the Na^+^,K^+^-ATPase β1 subunit has the unique ability, compared to the β2 and β3 subunits, to restore ZO-1, occludin, and VE-cadherin expression in previously LPS injured lungs. Lung injury was established in C57B6 mice (n = 6–8) by aspiration of LPS (5 mg/kg) and 1 day later plasmids (100 μg each) expressing either no insert (pcDNA3), or the β1, β2, or β3 subunits of the Na^+^,K^+^-ATPase (β1, β2, or β3) were delivered in 50 μl to the lungs by aspiration followed immediately by electroporation. Two days later, lungs were perfused with PBS and lysates were prepared for analysis by Western Blot (**A**). Levels of expression were normalized to GAPDH as a loading control and the relative expression of Occludin (**B**) and ZO-1 (**C**) are shown as mean ± SEM. All experiments were carried out three times and representative experiments are shown. One-way ANOVA with post-hoc Tukey’s multiple comparisons was used for statistical analysis; For Occludin (**B**) a, p < 0.01 compared to naïve; b, p < 0.001 compared to naïve; c, p < 0.05 compared to LPS alone; d, p < 0.01 compared to LPS + pcDNA3; e, p < 0.01 compared to LPS + β1; f, p < 0.05 compared to LPS + β1. For ZO-1 (**C**) a, p < 0.01 compared to naïve; b, p < 0.05 compared to LPS alone; c, p < 0.05 compared to LPS + pcDNA3. Full gels are in Fig. S3.

Tight junction complexes are critical in maintaining epithelial and endothelial barrier integrity^28^. They seal the lateral space of adjacent cells at the apical side, forming a tight barrier. Upon LPS assault, tight junction complexes are disrupted with downregulated protein expression. Our previous data showed that gene delivery of the β1 subunit into mouse lungs attenuated LPS increased lung permeability/leakage with significantly upregulated protein levels of tight junction proteins, (e.g., ZO-1 and occludin)^14^. Thus, we investigated whether delivering genes encoding the β2 or β3 subunit could restore ZO-1 and occludin protein expression in pre-injured mouse lungs. As described above, 1 day after LPS intratracheal administration, plasmids expressing β2- or β3-Na^+^, K^+^-ATPase were delivered by aspiration and electroporation. Then after 2 days, the lungs were harvested for western blots (Fig. 7). Compared to naïve (normalized to 1.000), levels of ZO-1 (0.721 ± 0.087) and occludin (0.721 ± 0.087) were decreased by up to three-fold in mice with LPS injury alone. There is no significant difference of protein quantitation between mice receiving LPS alone and LPS followed by pcDNA3. As seen previously, treatment of LPS pre-injured mice with β1 gene delivery significantly restored the protein levels of ZO-1 (0.634 ± 0.260, p < 0.05) and occludin (0.617 ± 0.159, p < 0.05), indicating a regulatory role of β1- Na^+^, K^+^-ATPase on the epithelial and endothelial barrier integrity. By contrast, gene transfer of β2-, or β3-Na^+^, K^+^-ATPase into the mouse lungs previously injured with LPS failed to upregulate ZO-1 (0.337 ± 0.047 for β2 mice, 0.320 ± 0.074 for β3 mice) or occludin (0.161 ± 0.059 for β2 mice, 0.242 ± 0.038 for β3 mice) levels, compared with groups with LPS injury alone or LPS and pcDNA3. We also have shown previously that gene transfer of the β1 subunit to cultured microvascular endothelial cells and to the mouse lung can increase expression of the adherens junction VE-cadherin^18^. Since we have shown that electroporation-mediated gene transfer can deliver genes to all cell types in the lung, including both the epithelium and endothelium, this suggests that the Na^+^,K^+^-ATPase β1 subunit signals similarly in endothelial cells and epithelial cells, as suggested in isolated cell studies^14,18^. Thus, we tested whether gene transfer of the β2 or β3 subunit could also increase VE-cadherin levels in these mice (Fig. 7). Whereas β1 overexpression led to significantly increased VE-cadherin levels, gene transfer of the β2 or β3 subunit failed to have any effect on levels of this protein. Collectively, the present data suggest that gene transfer of the β2 or β3 subunit of the Na^+^,K^+^-ATPase, while able to stimulate AFC to the same degree as the β1 subunit, were unable to repair multiple aspects of the disrupted or leaky lung barrier, including the decreased LPS induced pulmonary hyperpermeability, increased protein in BAL fluid, and decreased cell–cell adhesion tight and ahderens junction protein levels.

## Discussion

In this study, we tested whether gene delivery of the β2 or β3 subunit of the Na^+^, K^+^-ATPase could be used to alleviate injury and treat mouse lungs that had been previously injured with LPS, as we have shown previously with gene transfer of the β1 subunit^14,26^. Using electroporation to deliver plasmids encoding these genes to the lungs of mice, we found that overexpression of the β2 or β3 subunit significantly increased the alveolar fluid clearance compared to the basal level in naïve animals, indicating that the different isoforms could combine with the endogenously expressed α subunit and form functional Na^+^, K^+^-ATPase for ion transport. Moreover, the fact that the β2 and β3 subunits increased AFC to essentially the same degree as did overexpression of the β1 subunit suggests that all three subunits should have the same degree of therapeutic effect when delivered to the lungs of mice with existing lung injury if active alveolar fluid clearance is the driving force for disease treatment. However, we show that gene transfer of the β2 or β3 subunit into pre-injured animal lungs failed to attenuate the histological diffused alveolar damage, neutrophil infiltration, overall lung edema, or increased lung permeability, indicating that β2 or β3 gene delivery could not treat the LPS induced lung injury. Further, unlike the effect seen following β1 gene transfer in these animals, gene transfer of either the β2 or β3 subunits failed to have any effect on levels of tight or adherens junction proteins in the lungs and failed to improve lung barrier function, leading us to conclude that restoration of alveolar-capillary barrier function alone may be of equal or even more benefit than improving AFC for ALI/ARDS treatment.

Alveolar fluid clearance has been long studied as the major therapeutic goal for ARDS treatment since it was found that the majority of patients with ARDS showed severe fluid clearance impairment that was associated with prolonged acute respiratory failure and a higher mortality rate^29^. To this end, we and others have shown in animal models that if the initial pulmonary edema can be reduced by increasing AFC, inflammation and subsequent injury can also be lessened^7,26,30,31^. However, effective removal of pulmonary edema relies not simply on the ability to transport water out of the airspace, but also on the integrity of the alveolar-capillary barrier, thus keeping vascular and extravasated alveolar fluid out of the interstitium and airspaces. It is known that a net fluid clearance is based on an unimpaired AFC capacity and undisrupted alveolar capillary barrier which is composed of two physical barriers: a tight alveolar epithelial monolayer and a relatively more permeable microvascular endothelial monolayer. However, it is difficult to identify and compare the relative contributions of each of these two independent, yet intertwined, mechanisms for efficient edema resolution, since downregulated transepithelial ion transport due to injury is always associated with the alveolar barrier dysfunction, including paracellular junction disruption, epithelial cell apoptosis and necrosis.

Active ion transport-driven AFC is the primary mechanism for fluid clearance of cardiogenic edema and it has been demonstrated that β2-adrenergic activation of the cAMP pathway can enhance AFC through upregulation of the membrane abundance and activity of various ion transporters or channels, including the Na^+^,K^+^-ATPase, ENaC, and chloride channels^6,32^. In the case of the Na^+^,K^+^-ATPase, β2-adrenergic receptor agonists activate store operated calcium channels and recruit Na^+^,K^+^-ATPase-containing vesicles to the plasma membrane, thereby enhancing activity of the Na^+^ pump^11,12,33^. While β2 adrenergic agonists such as albuterol and salmeterol can treat ALI in mouse models through these mechanisms, they have failed to decrease the mortality rate or the number of ventilator-free days in clinical trials, despite showing marginal amelioration of pulmonary fluid accumulation in some trials^13^. These failures suggest that improvement of AFC alone may not be enough to correct ALI/ARDS pathology.

There are limited studies investigating the beneficial effects of re-establishing the alveolar capillary barrier for ARDS treatment, despite a clear understanding that both endothelial and epithelial barrier dysfunction play critical roles in the pathophysiology of the disease. In contrast, the breakdown of endothelial and epithelial barrier integrity clearly has been recognized as a key factor in the development of edema formation and establishment of ARDS^34^. For example, several groups have shown that the sphingosine 1-phosphate (S1P) receptor on the endothelial cell surface, when bound by S1P, enhances endothelial barrier integrity through actin cytoskeleton reorganization and localization of catenin and VE-cadherin molecules to the endothelial surface, supporting this as a potential intervention for ARDS^35,36^. In this respect, we have previously demonstrated that the treatment benefits gained by gene transfer of the β1 subunit of the Na^+^,K^+^-ATPase come not only from enhancing AFC by increasing ion transport activity, but also, more importantly, from restoring lung barrier function by increasing the abundance of tight and adherens junction proteins at the membrane, thereby decreasing lung permeability^14^. We identified the kinase MRCKα as a protein interactor with the Na^+^, K^+^-ATPase β1 subunit that is necessary for the junctional complex upregulation seen in both cells and in mouse lungs^18,19^. Upon overexpression of the β1 subunit, MRCKα is activated and in turn activates actin-myosin, circumferential actin bundling, and junctional recruitment via MLC2 phosphorylation^19^. Interestingly, neither the β2 or β3 subunits of the Na^+^, K^+^-ATPase interacted with MRCKα and both failed to stimulate overexpression of tight junction proteins in cultured type I alveolar epithelial cells^19^. When examined as a possible treatment for lung injury in mice, MRCKα gene transfer was indeed able to upregulate barrier function as in cultured cells and decrease injury to the same degree as β1 subunit gene transfer, but did not positively affect AFC^18^.

Our results suggest that the ion transport activity of the Na^+^,K^+^-ATPase (and hence AFC) is not required for the β1 subunit to regulate barrier function. We have found that even in the presence of oubain at concentrations shown to inhibit Na^+^,K^+^-ATPase ion channel and ATPase activity, overexpression of the β1 subunit in cultured ATI cells can still increase tight junction protein membrane abundance and transepithelial electrical resistance^19^. Further, we have shown that individual overexpression in mouse lungs of all of the β subunits increases AFC to a similar level, roughly twofold over that seen with naïve mice or those receiving the parental empty plasmid (Fig. 1). Further, others have shown that α1, the major alpha subunit expressed in the lung and other tissues, can effectively heterodimerize with β1, β2, or β3 subunits, all leading to enhanced Na^+^,K^+^-ATPase activity^23,37^. Since overexpression of β2 and β3 fails to upregulate tight and adherens junction protein levels and barrier function in cultured cells, if their gene transfer to LPS-injured lungs provided the same degree of treatment effect as does gene transfer of the β1 subunit, this would strongly favor a model in which upregulation of barrier function is not the primary mechanism of ALI/ARDS treatment by β subunit gene transfer. However, as we show here (Figs. 5, 6, and 7), this is not the case and neither β2 nor β3 overexpression, unlike that of the β1 subunit, has any treatment effect on lung injury, despite being able to enhance AFC to the same levels as with β1 overexpression (Fig. 1). This suggests that enhancement of AFC is not as important as strengthening or repairing alveolar-capillary barrier function in the treatment of LPS-induced ALI/ARDS.

Three isoforms of the β subunits are expressed in mammalian cells. Although there is isoform selectivity, all of them are able to bind to the α subunit, facilitating the correct folding, membrane recruitment and insertion of the α subunit and forming a functional ion transport complex^23,38^. However, sequence alignment shows that they have less than 25% sequence homology. Beyond acting as a chaperon of the α subunit, the β subunit of Na^+^, K^+^-ATPase has been shown to participate in many other cellular processes. The β1 isoform is widely expressed in mammalian cells and has been identified as a platform involved in various signal transduction pathways, and regulation of epithelial polarity, tight junction and paracellular barrier formation^39^. While the β1 is the most abundant β subunit in the lung, its targeted deletion in either type I and type II or only type I alveolar epithelial cells is not lethal, since compensatory upregulation of the β3 subunit is seen^40^. The β1 subunit is also involved in the cell–cell adhesion via its glycosylated extracellular domain (C-terminus)^41,42^, requiring the amino acid region 198–207 as well as N-glycosylation^23^. Interestingly, this region is lacking in both β2 and β3 subunits, and as such these β subunits fail to show homo- or heterodimer β-β interactions and cell–cell adhesive properties^39^. However, the β2 subunit has been shown to act as a cell adhesion protein, at least in glial cells^22,43^, although the mechanisms for this are unknown and may be limited to specific cell types. While β1-β1 trans-dimerization between cells has been suggested to be responsible for the formation and/or stabilization of epithelial adherens and tight junctions, clear mechanistic studies showing the relationship between the β1 trans-dimers and apical junctions are lacking^44,45^. There has been limited study investigating the non-transport function(s) of the β2 and β3 isoforms, especially in relation to intracellular junctions and barrier formation. However, our previously published data showed that overexpression of the β2 or β3 subunit failed to upregulate tight junction expression in vitro, indicating the role that is involved in epithelial barrier function is probably unique for the β1 subunit of the Na^+^, K^+^-ATPase.

Our results demonstrate that gene transfer of the β1 subunit of the Na^+^, K^+^-ATPase reduced multiple measures of lung injury but not to the levels seen in naïve animals. For example, a 27% decrease in pulmonary edema was observed when the β1 subunit was delivered compared to injury only, while a ~ 60% decrease in lung permeability and BAL protein or albumin content was observed. In none of these measures did the LPS-induced injury return to the pre-injury baseline. Several reasons could account for this. It is possible that the timing of outcome measures was not optimized to see maximal benefit, or that the amount of gene transfer and overexpression of the β1 subunit was insufficient to cause a complete reversal of the injury. Thus, it is possible that either delivering greater amounts of β1-expressing DNA or by repeat dosing of the DNA by electroporation-mediated gene delivery at some intervals could increase levels of the β1 transgene and further decrease measures of injury. Another approach could be to co-deliver other genes known to decrease inflammation or stimulate alveolar epithelial cell proliferation, both of which have shown some success in animal studies. For example, delivery HO-1 which has been shown to upregulate IL-10 levels or delivery of IL-10 directly has been shown to decrease lung injury, by mechanisms unrelated to fluid clearance or barrier repair^46–48^. Similarly, delivery of keratinocyte growth factor (KGF) promotes type II cell proliferation and partially protects from lung injury^49^. While none of these approaches have provided complete protection from subsequent lung injury, it is possible that a combinatorial approach in which multiple distinct pathways are targeted could provide even greater benefit that we have seen here in our treatment studies.

In conclusion, our data support a model in which stimulation of alveolar fluid clearance alone is insufficient to attenuate previously existing lung injury caused by LPS aspiration in mice and point to repair or strengthening of the alveolar-capillary barrier by upregulation of intercellular junctional complexes as being needed for effective treatment. These studies support a growing body of data suggesting that repair of barrier function is necessary for any meaningful alveolar fluid clearance to occur that can lead to lessening of the disease^34^. Taken together with results from multiple clinical trials demonstrating that upregulation of alveolar fluid clearance alone has no positive benefit in patients^13^, these results may have lasting impact on development of treatment strategies for ALI/ARDS in patients.

## Supplementary Information

Supplementary Figures.

## Data Availability

All data generated or analyzed during this study are included in this published article.
