# Correction: Upregulation of alveolar fluid clearance is not sufficient for Na^+^,K^+^-ATPase β subunit-mediated gene therapy of LPS-induced acute lung injury in mice

**DOI:** 10.1038/s41598-026-50284-w

**Published:** 2026-05-04

**Authors:** Jing Liu, Gillian M. Schiralli‑Lester, Rosemary Norman, David A. Dean

**Affiliations:** 1 Department of Pediatrics, University of Rochester, 601 Elmwood Avenue, Box 850, Rochester, NY 14642 USA; 2 Department of Pharmacology and Physiology, University of Rochester, 601 Elmwood Avenue, Rochester, NY 14642 USA

Correction to: *Scientific Reports* 10.1038/s41598-023-33985-4, published online 26 April 2023

This Article contains errors.

As a result of errors during figure assembly, in Figure 3A the first image for LPS + pcDNA3 was a duplication of the second image for LPS. In Figure 3A, the second image for LPS + pcDNA3 was a duplication of the third image for LPS. In Figure 3B, the third image for LPS was a duplication of the first image for LPS + pcDNA3. In Figure 3B, the third image for LPS + β1 was a duplication of the first image for LPS + β3.

Additionally, during the review of the data in preparation for this Correction, the Authors found out that in Figure 3A, the fourth image for LPS + β3 originated from a different animal. All these images were replaced with the correct data.

The correct Figure 3 and its accompanying legend appear below.

**Fig. 3:**
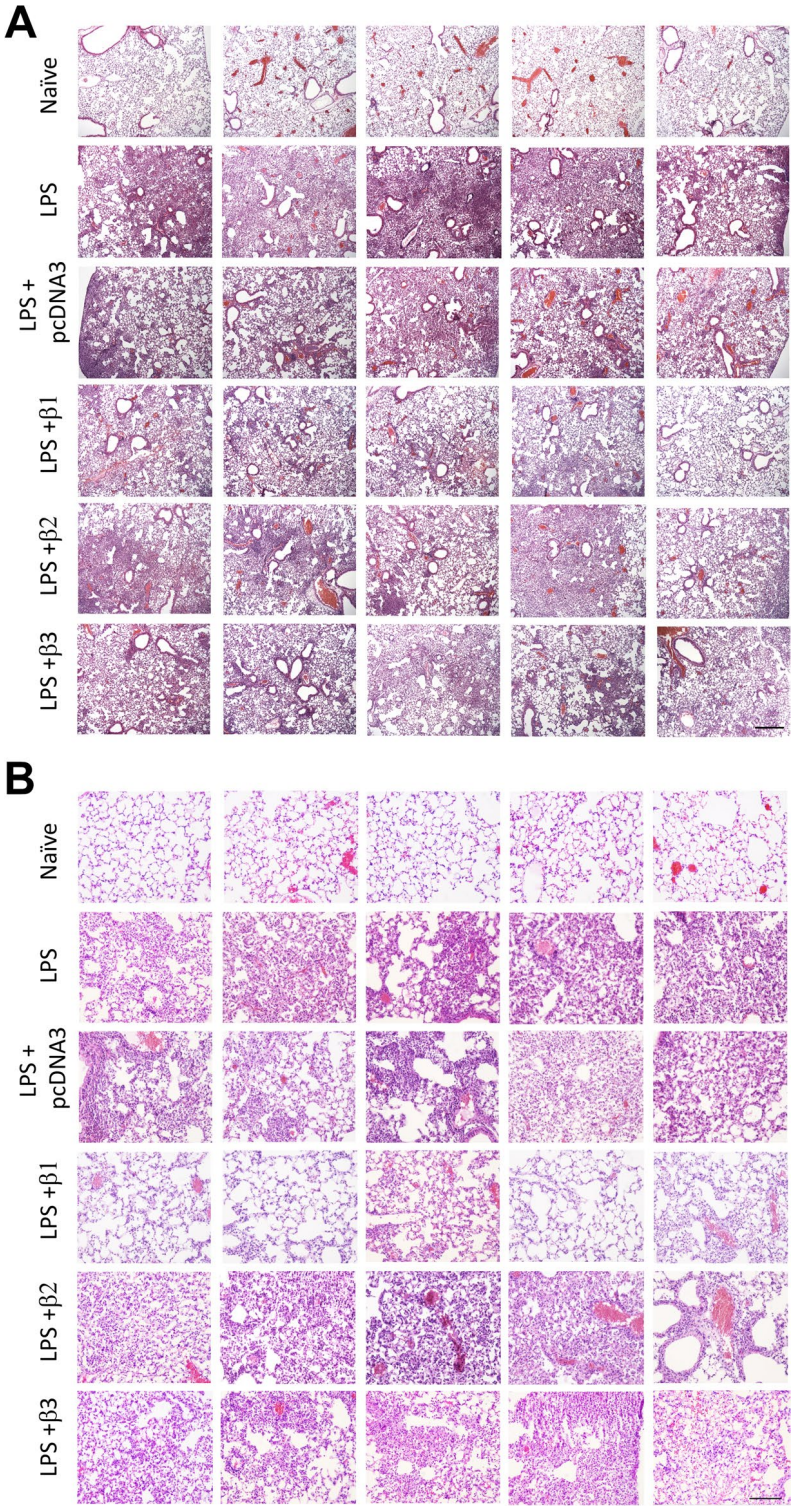
Overexpression of the β2 or β3 subunits of the Na^+^,K^+^-ATPase fails to attenuate LPS-induced lung injury. Lung injury was established in mice (n = 6–8) by aspiration of LPS (5 mg/kg) and 1 day later plasmids (100 μg each) expressing either no insert (pcDNA3), or the β1, β2, or β3 subunits of the Na^+^,K^+^-ATPase (β1, β2, or β3) were electroporated into lungs as in Fig. 2. Naïve mice (n = 5) received no LPS or DNA. Two days after electroporation (3 days after LPS administration), lungs were inflated to 20 cm H_2_O with 10% buffered formalin and processed for paraffin-embedding, sectioning, and hematoxylin and eosin staining. Sections from 5 representative animals are shown at ×50 (**A**) and ×400 (**B**) magnification. All experiments were carried out three times and a representative experiment is shown. Scale bar is 300 μm (**A**) and 100 μm (**B**).

