# Supplementary material for: Upregulation of alveolar fluid clearance is not sufficient for Na+,K+-ATPase β subunit-mediated gene therapy of LPS-induced acute lung injury in mice
Source: Sci Rep. 2023 Apr 26;13:6792. doi: 10.1038/s41598-023-33985-4 (PMC10130817; doi:10.1038/s41598-023-33985-4)
Supplement: Supplementary file 1 — Supplementary Figures. [file 41598_2023_33985_MOESM1_ESM.docx]

**Supplementary Information**

**Figure S1. Distribution of Evans Blue Dye following oropharyngeal aspiration of fluid in mouse lungs.** Fifty microliters of Evans Blue Dye-labeled albumin in saline were delivered to the lungs of lightly anesthetized mice (n=5) by aspiration. Animals were euthanized and less than 5 minutes after aspiration, the lungs and esophagus/stomach were removed from the animals, dried for 24 hours at 60°C, and then formamide-extracted dye was quantified spectrophotometrically. P<0.01 by Man-Whitney U test.


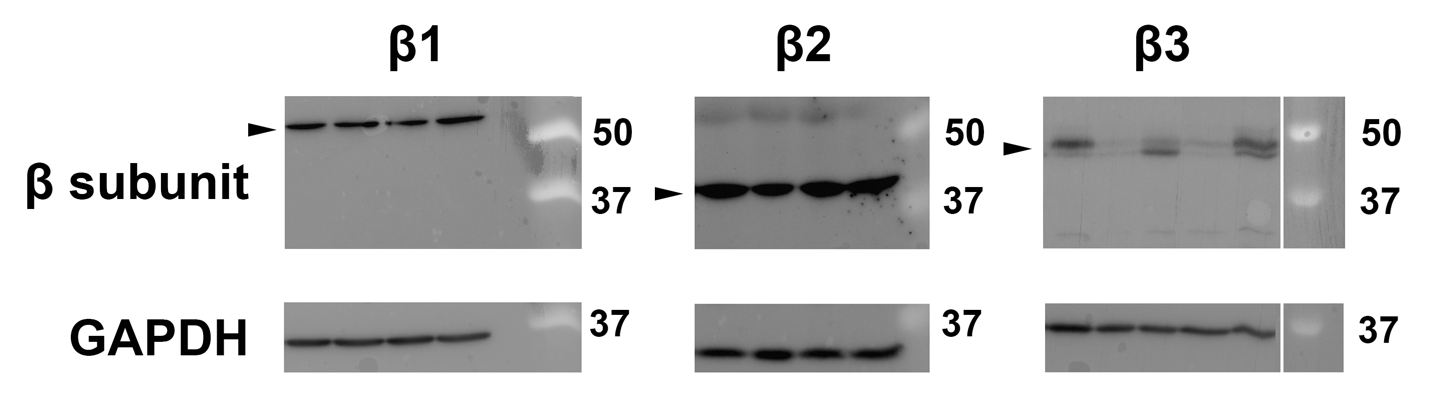


**Figure S2. Expression of β1, β2, and β3 subunit expression in the lungs of mice following electroporation-mediated gene delivery.** Plasmids (100 µg) expressing DDK-tagged β1, β2, or β3 subunits of the Na^+^,K^+^-ATPase were delivered to the lungs of C57B6 mice in 50 µl of 10 mM Tris, pH8, 1 mM EDTA, and 140 mM NaCl by aspiration followed immediately with 8 square wave pulses of 10 msec duration each at a field strength of 200 V/cm. Two days later, lungs were perfused, excised, and lysates were prepared for SDS-PAGE and western blot analysis. Equal quantities (8 µg/lane) of protein were loaded in each lane and blots were probed using anti-DDK or anti-GAPDH antibodies followed by appropriate secondary antibodies and chemiluminescent substrate. Protein expression in the lungs from 4 to 5 representative animals are shown for each subunit. Arrows denote the predicted size of the beta subunits and molecular weight markers are shown on the left). Full gels are in Fig. S3.


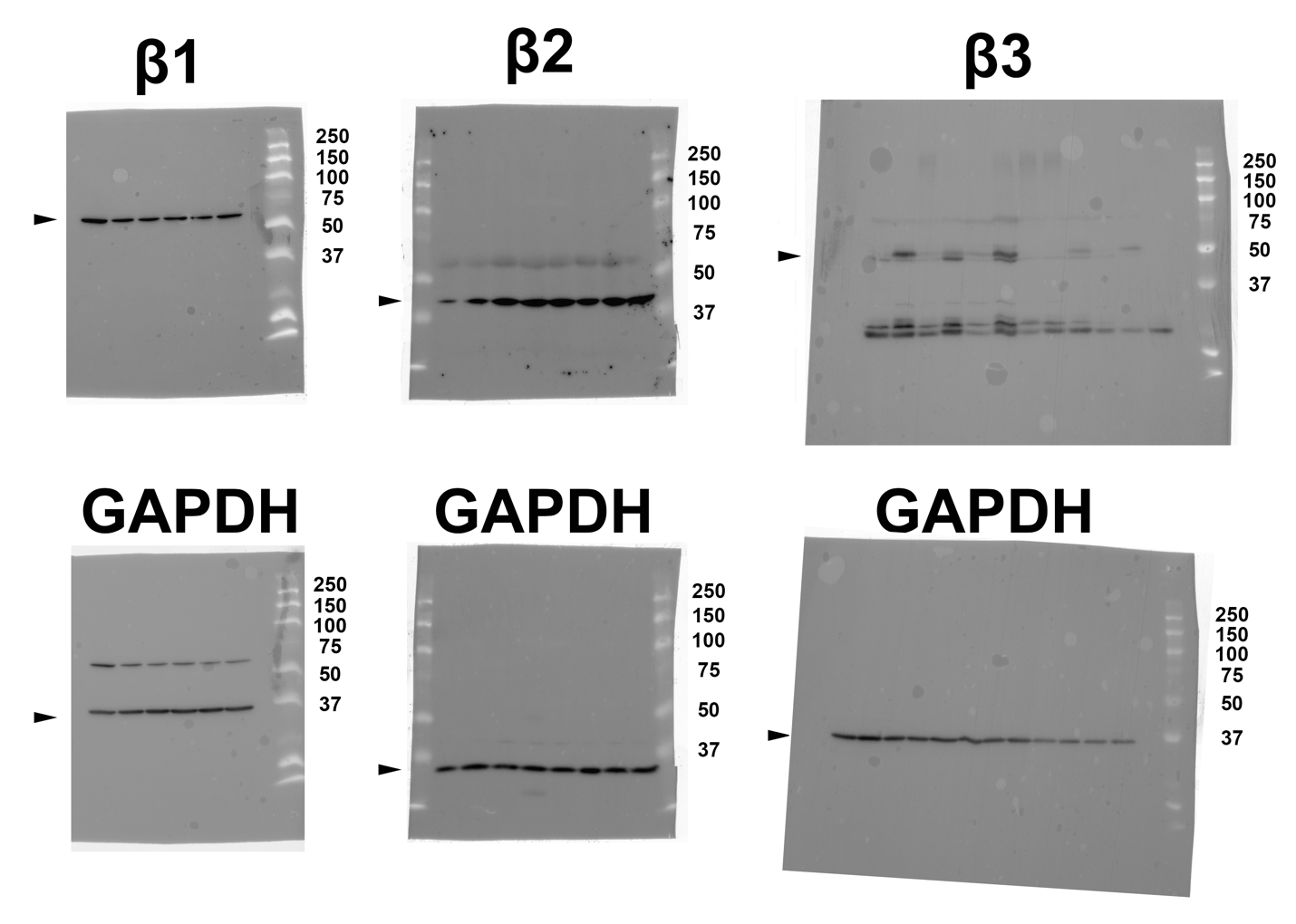


Fig. S.3. Full gels/Western blots for Figure S2. Molecular weight markers are shown at right and arrows indicate the correct band size for the respective protein. Each lane is from a different animal that had received the respective beta subunit plasmid by electroporation.


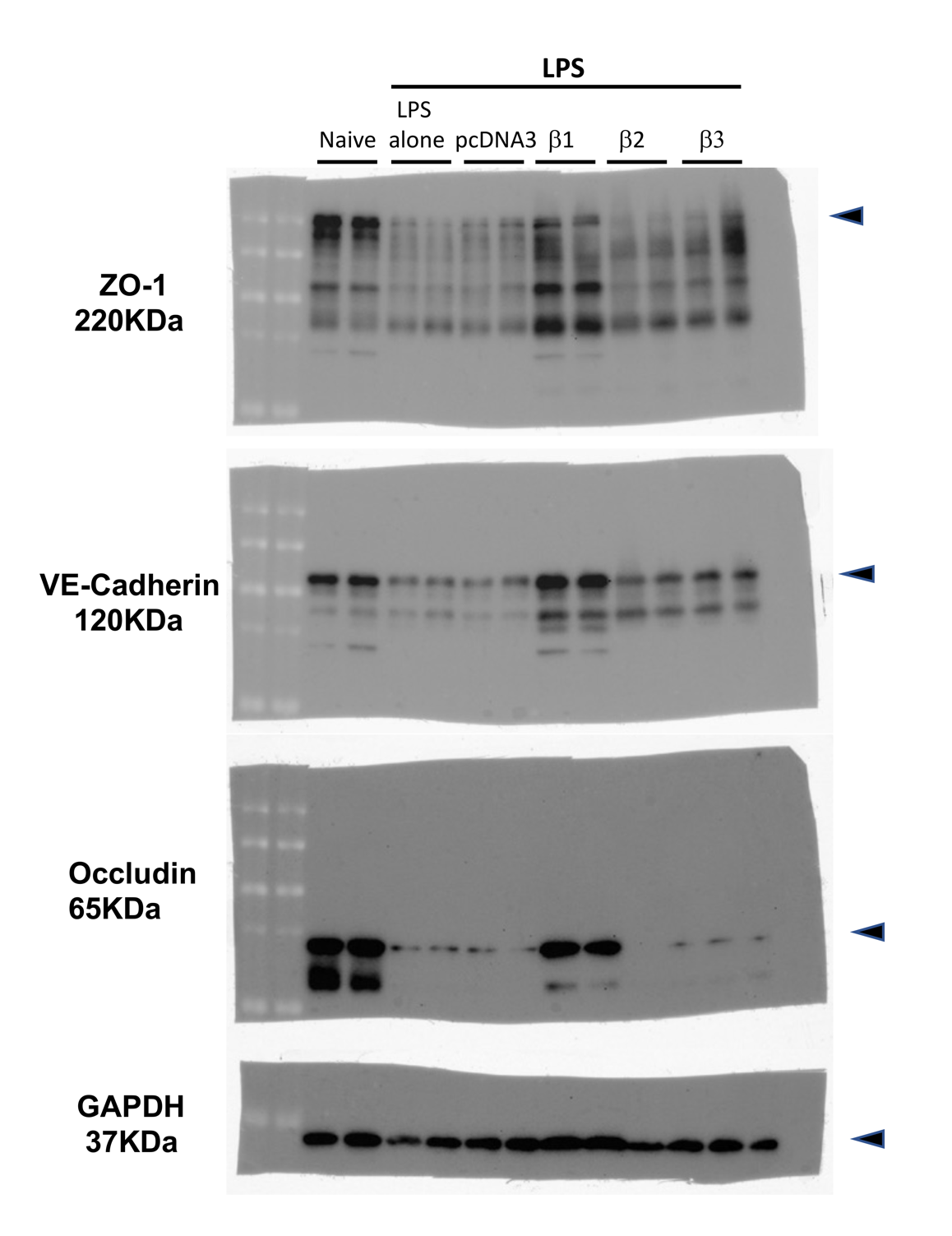


Figure S4. Full gels/Western blots for Figure 7. Molecular weight markers are shown at right and arrows indicate the correct band size for the respective protein.
